# Porous dendritic copper: an electrocatalyst for highly selective CO_2_ reduction to formate in water/ionic liquid electrolyte†
†Electronic supplementary information (ESI) available. See DOI: 10.1039/c6sc03194c


**DOI:** 10.1039/c6sc03194c

**Published:** 2016-09-20

**Authors:** Tran Ngoc Huan, Philippe Simon, Gwenaëlle Rousse, Isabelle Génois, Vincent Artero, Marc Fontecave

**Affiliations:** a Laboratoire de Chimie des Processus Biologiques , CNRS UMR 8229 , Collège de France , Université Pierre et Marie Curie , 11 Place Marcelin Berthelot , 75231 Paris Cedex 05 , France . Email: mfontecave@cea.fr ; Tel: +33 0144271372; b Université Grenoble Alpes , 38000 Grenoble , France . Email: vincent.artero@cea.fr; c Laboratory of Chemistry and Biology of Metals , CNRS UMR 5249 , 17 rue des Martyrs , 38054 Grenoble , France; d Commissariat à l'énergie atomique et aux énergies alternatives (CEA) , Fundamental Research Division , 38000 Grenoble , France; e Laboratoire Chimie du Solide et Energie , CNRS FRE 3677 , Collège de France , Université Pierre et Marie Curie , 11 Place Marcelin Berthelot , 75231 Paris Cedex 05 , France; f Laboratoire de Chimie de la Matière Condensée de Paris , Collège de France , 11 place Marcelin Berthelot , 75005 Paris , France

## Abstract

Copper is currently extensively studied because it provides promising electrodes for carbon dioxide electroreduction. The original combination, reported here, of a nanostructured porous dendritic Cu-based material, characterized by electron microcopy (SEM, TEM) and X-ray diffraction methods, and a water/ionic liquid mixture as the solvent, contributing to CO_2_ solubilization and activation, results in a remarkably efficient (large current densities at low overpotentials), stable and selective (large faradic yields) electrocatalytic system for the conversion of CO_2_ into formic acid, a product with a variety of uses. These results provide new directions for the further improvement of Cu electrodes.

## Abstract 2

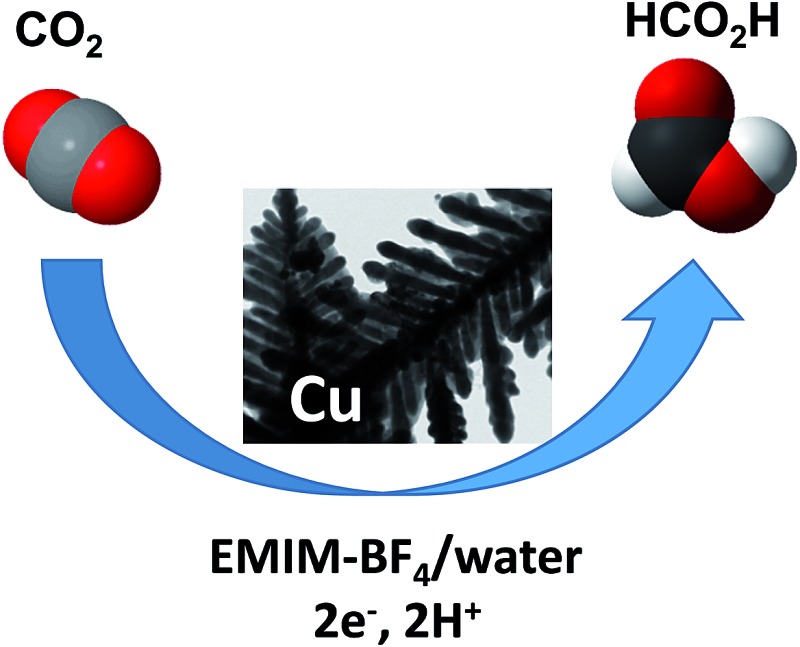
An ionic liquid/water electrolyte promotes excellent selectivity for CO_2_ electroreduction to formic acid at a porous dendritic copper electrode material.

## Introduction

Although reduction of CO_2_ into energy-dense liquids or gaseous fuels is a fascinating strategy in the context of global warming and substitution of renewable energies for fossil fuels, its practical implementation is highly challenging due to the stability of the CO_2_ molecule. Furthermore, CO_2_ transformation can generate a great variety of compounds but these reactions also involve multiple electrons and protons. Another recurring and crucial issue is competition with proton reduction into dihydrogen. Thus the development of selective and efficient catalysts and the appropriate tuning of reaction conditions (temperature, pressure, solvent, *etc.*) are essential.^1^

Regarding catalysts for CO_2_ reduction, current research mainly focuses on coordination complexes under homogeneous conditions or on solid-state, mostly metallic, electrodes.^2–4^ Following pioneering reports from Hori,^5,6^ various metals with different structures (nanoparticles, nanorods, dendrites, *etc.*) have been carefully revisited as electrocatalysts for CO_2_ conversion with special attention paid to electrodes based on abundant and cheap non-noble metals.^7–10^ Among them, Cu is of specific interest as it is low cost and has a high catalytic potential for transforming CO_2_ into various products including methane and hydrocarbons.^9,11–21^ However, these systems still have a major drawback of being relatively unselective, generating a complex mixture of products often including a large proportion of hydrogen.^13^ Selectivity is a key issue for technological applications as it facilitates product separation. There is therefore a need to develop more selective Cu-based catalysts. For example, while Cu catalyzes the electroreduction of CO_2_ to formate, faradic yields never exceed 40%.^9,19,22^

In the future conversion of CO_2_ to formic acid may indeed have a number of useful industrial applications.^23,24^ Traditional uses for formic acid have been in the leather tanning and animal feed markets. It can also be used to replace mineral acids. Furthermore it is a suitable H_2_ storage material as it can be easily and selectively decomposed into H_2_ and CO_2_ in the presence of a catalyst.^24^ At ambient temperature and pressure formic acid stores 580 times more H_2_ than the same volume of gas.^23^ Formic acid can also be converted into syngas.^23^ Finally formate salts are used as effective, non-corrosive and environmentally friendly anti-icing agents. Development of new and sustainable technologies that would decrease the cost of formic acid production might lead to an increased demand which is currently still low (500 000 t yr^–1^).^23^

Recently, CO_2_ electroreduction in ionic liquids (ILs) has been a matter of interest.^25–29^ In 2011 Rosen *et al.* first demonstrated stable production of CO from CO_2_-saturated H_2_O/IL solvent with high faradic yield and a very low overpotential using a silver cathode.^30^ Following this observation several metal-based electrodes known to catalyze CO_2_ electroreduction have been reevaluated under comparable conditions, with the notable exceptions of gold and copper.^31–33^ ILs are redox-robust, generally possess a wide electrochemical window and are better than water and organic solvents for solubilizing CO_2_.^34^ Interestingly, the imidazolium cations of ILs can act as co-catalysts during CO_2_ reduction by stabilizing catalytic intermediates.^35,36^ This interaction lowers the activation barrier, therefore significantly reducing the overpotential requirement for the reduction of CO_2_.

Herein, we report that a porous and dendritic Cu-based material,^37,38^ with high specific surface area, displays high selectivity for formic acid production when assessed in an ionic liquid electrolyte, 1-ethyl-3-methylimidazolium tetrafluoroborate, [EMIM](BF_4_), in the presence of water. Such an activity is sustained for hours at high current densities at a moderate operational potential without deactivation of the electrode. A comparable material has been investigated for electroreduction of CO_2_ in aqueous conditions but gave low faradic yields (<40%) of CO_2_ reduction products, with H_2_ being the major product.^11,19^ Given the general versatility of copper electrodes for producing a variety of CO_2_ reduction products, our study highlights the importance of the media to orient the reaction. Importantly ionic liquids specifically favor CO_2_ reduction to formate over H_2_ evolution at copper electrodes, while more complex mixtures are obtained when using aqueous electrolytes.

## Results and discussion

Electrodeposition of Cu from an acidic CuSO_4_ solution was carried out on a Cu plate electrode. Application of a large current (0.5 A cm^–2^) during a short period of time (20–120 s) resulted in the intense formation of hydrogen bubbles at the Cu plate electrode which contributed to Cu deposition in the form of a nanostructured foam, consisting of three-dimensional Cu porous dendrites, as shown by electron microscopy (see below).

**Fig. 1:**
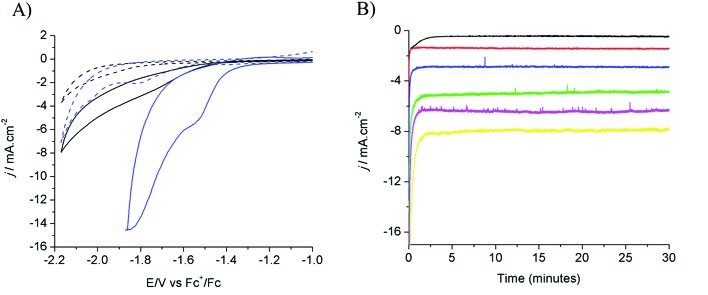
(A) Cyclic voltammograms recorded on a modified Cu electrode (80 s electrodeposition) (blue) and Cu plate electrode (black) in [EMIM](BF_4_)/H_2_O (92/8 v/v) after N_2_ purging (dashed lines) and CO_2_ purging (solid lines). (B) Current densities during electrolysis in CO_2_-saturated [EMIM](BF_4_)/H_2_O (92/8 v/v) at different potentials: –1.25 V (black), –1.35 V (red), –1.45 V (blue), –1.55 V (green), –1.65 V (magenta), and –1.75 V (yellow) *vs.* Fc^+^/Fc.

**Fig. 2:**
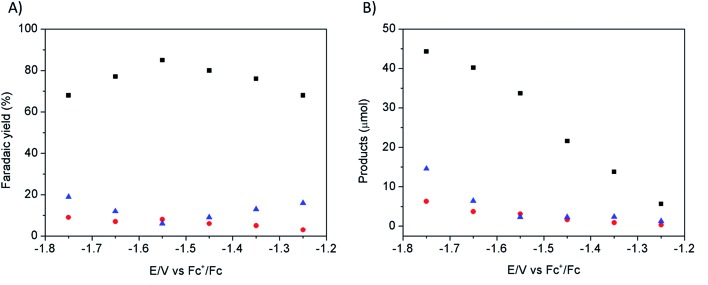
CO_2_ reduction products recorded on a modified Cu electrode (80 s electrodeposition) as a function of applied potential: faradic yields (A) and absolute amounts (B) of formate (black squares), CO (red circles) and H_2_ (blue triangles) during 30 minutes electrolysis in CO_2_-saturated [EMIM](BF_4_)/H_2_O (92/8 v/v).

Electrochemical characterization of the electrode obtained after 80 s electrodeposition was carried out by cyclic voltammetry (CV) and controlled potential electrolysis (CPE), using a [EMIM](BF_4_)–water mixture (92/8 v/v) as the electrolyte. Fig. 1A compares the CVs obtained with a standard Cu plate and the modified electrode, either under N_2_ or at CO_2_ saturation. A catalytic wave was observed in both cases upon addition of CO_2_. However the modified electrode gave much larger current densities (values are normalized with respect to the geometry surface area, 1 cm^2^) at any potential. The catalytic wave, with an onset potential of –1.2 V *vs.* Fc^+^/Fc, displayed a peak at –1.5 V *vs.* Fc^+^/Fc followed by a second peak at –1.8 V *vs.* Fc^+^/Fc, likely reflecting two different CO_2_ reduction mechanisms whose identification requires further investigation. A comparable behavior has previously been reported for an indium disc electrode.^33^ Fig. 1B shows the effect of the applied potential on the current density during CPE using the same modified electrode. Increased current densities were observed as a more negative potential was applied. Product analysis showed formation of formate, CO and H_2_ with excellent overall faradic yields (85–95%) (Fig. 2). The best selectivity for the formation of CO_2_ reduction products was achieved with an applied potential of –1.55 V *vs.* Fc^+^/Fc with a total faradic yield of almost 90% (formate: 83%; CO: 6%). It was confirmed that this formate and CO originated from CO_2_ by ^13^C-NMR and mass spectrometry analysis of the CPE experiment reaction mixture using ^13^CO_2_ as the substrate (Fig. S1†). The same experiment has been carried out using a Cu plate electrode. In this case, we could not achieve more than 45% formate (3% CO) formation, however this experiment was conducted at a much more negative potential (–1.8 V *vs.* Fc^+^/Fc) and with a much lower current density (1.8 mA cm^–2^). The major product was H_2_.

The effect of the electrodeposition time on the activity of the modified electrode was studied by CPE at –1.55 V *vs.* Fc^+^/Fc at CO_2_ saturation in [EMIM](BF_4_)/H_2_O (92/8 v/v). As shown in Fig. S2,† increased activity was obtained upon increasing the electrodeposition time (current intensity values of 6.5 mA cm^–2^ for 80 s electrodeposition as compared to 4 mA cm^–2^ for 40 s electrodeposition). For both 40 s and 80 s electrodeposited samples, faradic yields were above 90% with formic acid (83%) as the major product and only small amounts of CO and H_2_ (see Table S1†). A longer deposition time (120 s) had only a minor effect on the current density but resulted in a slightly larger production of H_2_ (Table S1†). As a control experiment, electrolysis under N_2_ saturation gave a current density of only 0.5 mA cm^–2^ (Fig. S2†).

The effect of water concentration was studied by CV and CPE in [EMIM](BF_4_)/H_2_O (85/15 v/v) (Fig. S3†). Under these conditions, much higher current densities correlated with a larger production of H_2_, with formate accounting for only 50% of the products (Table S2†).

Finally the modified electrode proved to be stable during long-term electrolysis as shown from an 8 hour experiment (current density: 6.5 mA cm^–2^. Faradic yields for formate: 87%; CO: 5%; H_2_: 7%) (Fig. S4†).

Further evidence for the 80 s electrodeposition providing an optimized electrode is presented in Fig. S5 and S6.† This evidence shows that the Cu reduction peak observed at –0.05 V *vs.* Ag/AgCl increases as a function of the electrodeposition time but levels off almost after 80 s (Fig. S5A†). The amount of surface-active copper, calculated from Fig. S5A,† is at least 7 times larger than that of a standard Cu plate electrode (Fig. S5B†). In addition the Randles–Sevcik equation (ESI†), when applied to the reduction of 5 mM K_3_[Fe(CN)]_6_ (Fig. S6†), was used to estimate the electrochemical diffusion surface area of both the Cu plate and the modified Cu electrode (80 s electrodeposition), giving *A*_diff_ = 2.04 cm^2^ and 19.8 cm^2^, respectively.

**Fig. 3:**
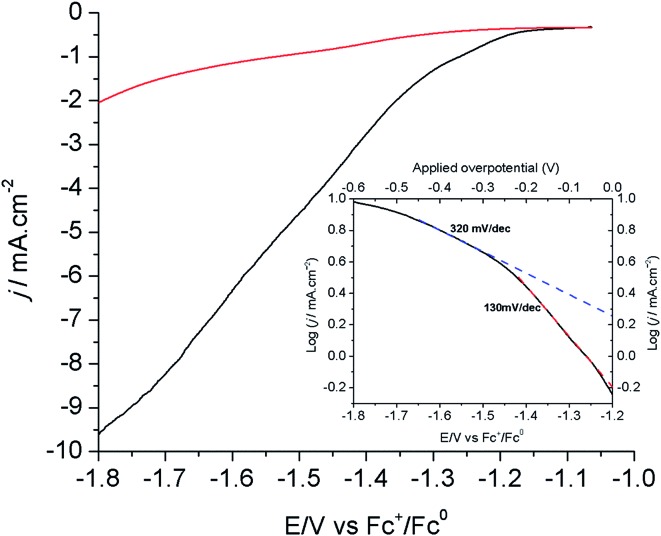
LSVs of the modified Cu electrode (80 s electrodeposition) in N_2_- (red) and CO_2_-saturated (black) [EMIM](BF_4_)/H_2_O (92/8 v/v). Inset: Tafel plot for the electrode in CO_2_-saturated [EMIM](BF_4_)/H_2_O (92/8 v/v).

Fig. 3 shows the linear sweep voltammograms (LSVs) of the modified Cu electrode (80 s electrodeposition) at a scan rate of 1.0 mV s^–1^ in CO_2_- and N_2_-saturated [EMIM](BF_4_)/H_2_O (92/8 v/v) solutions. As shown above, the current measured under these conditions mostly corresponds to the reduction of CO_2_ into formic acid. The corresponding Tafel plot is shown in the inset of Fig. 3. The onset potential for this reaction is –1.2 V *vs.* Fc^+^/Fc, in good agreement with the CV measurements shown in Fig. 1A. The Tafel data are linear in the range of –1.2/–1.5 V *vs.* Fc^+^/Fc, with a slope of 130 mV per decade, a value consistent with a mechanism in which the first electron transfer to adsorbed CO_2_ is the rate determining step.^12^ At a more negative potential, the slope significantly increases to 300–400 mV per decade. By analogy with oxygen reduction reaction studies at porous electrode materials, this is indicative of current limitation by mass transport phenomena including both CO_2_ diffusion and proton migration within the porous material dipped in the electrolyte.^39^ Under such conditions, current densities already exceed 10 mA cm^–2^.

The evaluation of overpotential values is not trivial here as the equilibrium potential of the CO_2_/HCOOH couple in [EMIM](BF_4_)/H_2_O is unknown. However, we determined by LSV the onset potential for electroreduction of CO_2_ in CH_3_CN/H_2_O (92/8 v/v) + 0.1 M *n*-Bu_4_BF_4_ at –1.4 V *vs.* Fc^+^/Fc (Fig. S7†). The equilibrium potential of the CO_2_/HCOOH couple in CH_3_CN is –1.32 V *vs.* Fc^+^/Fc when considering hydrated CO_2_ as the strongest acid in solution (ESI†). Under these conditions, the modified Cu electrode shows a remarkably low overpotential requirement of 80 mV. In addition, shifting from the organic solvent to the IL/water solvent resulted in an ∼200 mV decreased onset potential. Similarly, IL-promoted decrease of the onset potentials has been observed at Ag-based electrodes and have been related to the interaction of the IL with CO_2_ and/or with the surface during the catalytic transformation resulting in decreased activation barriers.^26,30,40^ As the onset for catalysis is observed at –1.2 V *vs.* Fc^+^/Fc in [EMIM](BF_4_)/H_2_O (92/8 v/v), we conclude that the equilibrium potential in this solvent is more positive than in CH_3_CN. This may result from various factors including a stronger solvation energy of formic acid and a lowered p*K*_a_ value of hydrated CO_2_ acting as the proton source.

**Fig. 4:**
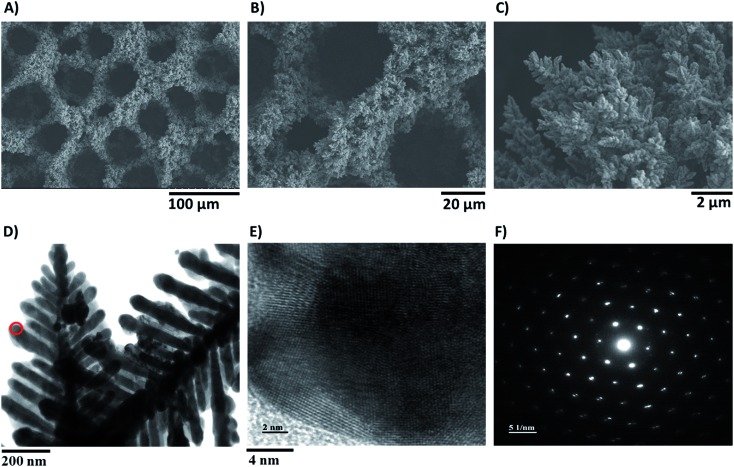
(A–C) SEM images of the modified Cu electrode (80 s electrodeposition) at different magnifications; (D) TEM image of a Cu dendrite; (E) atomic resolution image taken from the area indicated (red circle) in (D); selected area electron diffraction (SAED) pattern of a Cu nanodendrite (F).

In order to get some insights into the structure of the modified active electrodes, electron microscopy and X-ray diffraction (XRD) analysis were carried out. Scanning electron microscopy (SEM) images of the modified electrodes were obtained after different deposition times (20 s, 40 s, 60 s, 80 s, 120 s, and 160 s) at a constant applied current of 0.5 A cm^–2^ (Fig. 4A–C and S8†). They revealed a porous structure with pores having a diameter of 30–40 µm and the presence of a network of dendrites forming the walls within the Cu foam. Fig. S8† shows that the porosity and the nanostructure of the Cu electrodes are dependent on the deposition time, with an optimized porosity achieved after an 80 s or 120 s electrodeposition time, in agreement with the observed effect of deposition time on catalytic activity.

A transmission electron microscopic (TEM) image of the Cu dendrites is shown in Fig. 4D, and a high resolution image allows clear observation of the Cu atomic structure (Fig. 4E). A selected area electron diffraction (SAED) image of these dendrites is shown in Fig. 4F, in which a circular diffraction pattern indicated a highly crystalline structure.

**Fig. 5:**
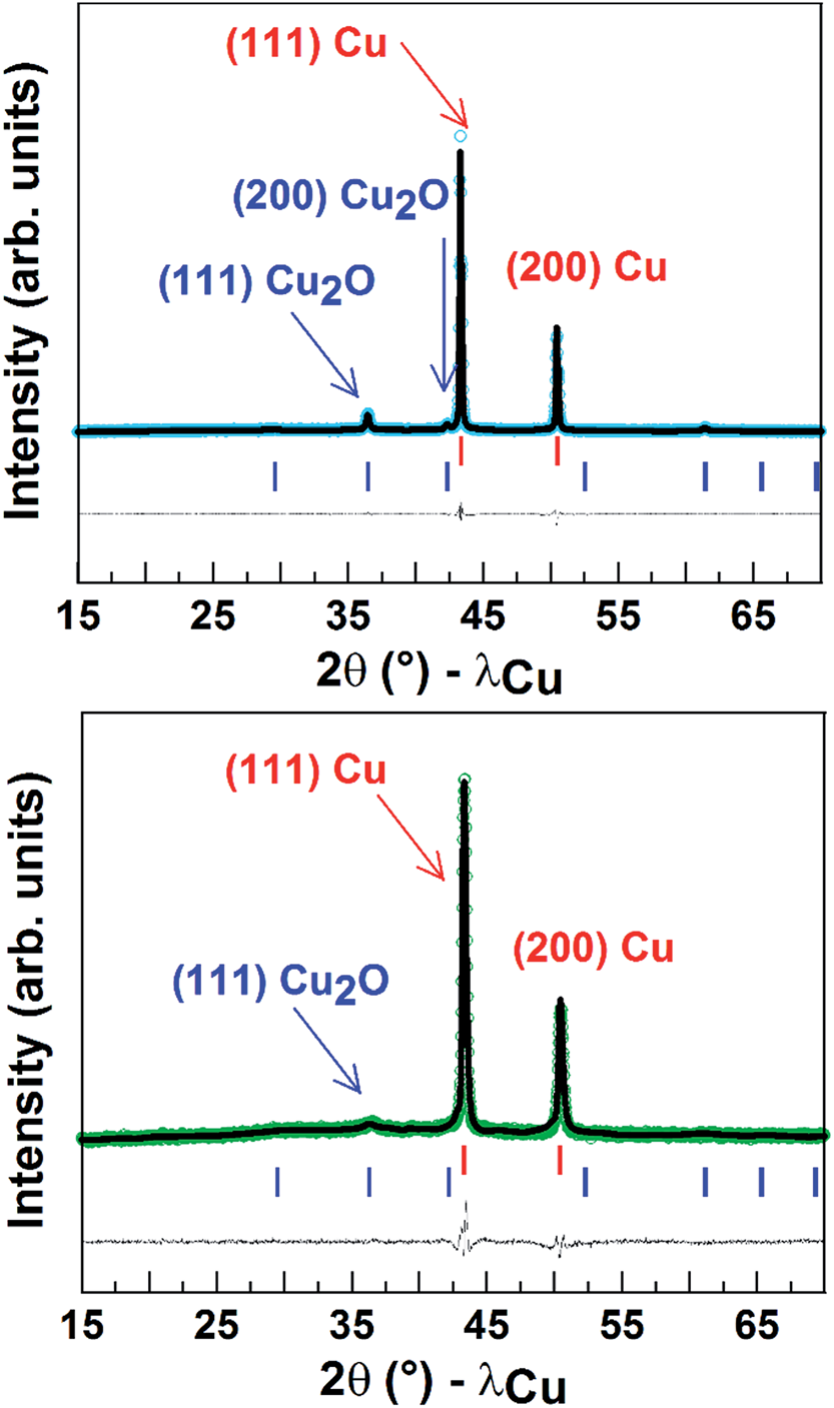
Rietveld refinement of X-ray patterns of a powder of the Cu porous material (80 s electrodeposition) before (top) and after (below) CO_2_ electroreduction. The observed (open circles), calculated (black line) and difference (blue line on top and green line on bottom) patterns are shown. The red and blue vertical tick bars represent the Bragg positions for Cu and Cu_2_O, respectively.

Finally, Fig. 5 shows the powder X-ray diffraction patterns obtained for the Cu porous material which indicated a major contribution from Cu^0^. The spectrum also displays a small peak corresponding to Cu_2_O and accounting for around 10 (±2)% of the mass. After 1 h CO_2_ electroreduction, XRD analysis confirmed that the electrode material has kept its crystalline state, while the intensities of the peaks corresponding to Cu_2_O significantly decreased (5 ± 2%) in agreement with reduction of Cu^I^ to Cu^0^ during the reaction.^9^ As shown from the SEM images taken after long-term CPE, the Cu material still displayed a porous and dendritic structure (Fig. S9†). Furthermore the amount of surface active copper after catalysis (0.74 µmol cm^–2^) is similar to that of the same electrode before catalysis.

## Conclusion

We here show that a low cost Cu porous dendritic material, as shown by SEM, TEM and XRD, displays remarkable electrocatalytic activity for the reduction of CO_2_. It is a unique electrocatalyst as it is the first copper-based material for the catalytic conversion of CO_2_ into formic acid in a H_2_O/IL solvent with such a high faradic yield. The selectivity for formate production is the highest reported so far at low overpotentials for a Cu-based electrode. As a representative example, Cu nanoparticles, developed by Kanan *et al.*, afforded only 33% formic acid under electrolysis at an overpotential of 250 mV with a current density of 5 mA cm^–2^ in aqueous KHCO_3_ solution. Formic acid can be obtained with a few other metallic electrodes in IL/water media. For example In and Sn electrodes are classically known as selective catalysts for formate production in aqueous media and retain their high selectivity (90%) in ionic liquid/water electrolytes, but with low current densities (<2 mA cm^–2^) and only at very negative potentials (–1.95 V *vs.* Ag/AgCl, 3 M NaCl).^33^ Furthermore at lower potentials the faradic yield drastically decreased as a result of H_2_ formation becoming predominant. During the course of the revision of this article a selective reduction of CO_2_ to formate (faradic yield: 90%) using Pb and Sn electrodes was reported.^41^A very high current density is reported, but only at extremely negative potentials, at least 300 mV more negative than those operating in the system we report here, and only using very complex solvent mixtures including ILs, water and an organic solvent. Recently a high faradic yield for formic acid, at a low overpotential was obtained using a superbase ionic liquid, however with lower current densities and using an electrode based on silver.^42^

The increased specific active surface of our Cu electrode provided by the dendritic porous network combined with high CO_2_ solubility in ILs likely explains the high current densities (>10 mA cm^–2^) observed during electrolysis. The large selectivity for formic acid is more intriguing. Indeed, Cu electrodes are well known to produce hydrocarbons but at very negative potentials and to produce hydrogen at more positive potentials in aqueous solutions.^5,6^ Indeed, the ability of Cu to reduce protons has recently been supported by theoretical and experimental studies.^43,44^ It is tempting to suggest that the IL specifically contributes to activate CO_2_ enough so as to reorient the reactivity of the activated H atoms towards CO_2_ to produce formate. The low production of H_2_ and CO at low overpotentials make the system reported here unique and these characteristics are likely to be explained by the combination of the nanodendritic porous structure of the catalytic material and the use of the ionic liquid/water mixed electrolyte, illustrating the importance of the reaction medium. As a matter of fact, during the preparation of this article, a similar porous nanodendritic Cu material was reported and investigated under different solvent and electrolytic conditions. It was shown to produce a more complex mixture of products, with much larger amounts of H_2_ and CO and less formate, but, more interestingly, with significant amounts of ethane and ethylene (no methane) with efficiency for C2 products reaching 55%.^11^ This porous dendritic Cu material thus seems to show great potential as a platform for the development of even more efficient and selective Cu-based systems, as selectivity is a major issue with respect to technological applications.
