# Supplementary material for: Porous dendritic copper: an electrocatalyst for highly selective CO2 reduction to formate in water/ionic liquid electrolyte
Source: Chem Sci. 2016 Sep 20;8(1):742–7. doi: 10.1039/c6sc03194c (PMC5299793; doi:10.1039/c6sc03194c)
Supplement: Supplementary file 1 [file SC-008-C6SC03194C-s001.pdf]

Electronic Supplementary Material (ESI) for Chemical Science.
This journal is © The Royal Society of Chemistry 2016

Porous dendritic copper: an electrocatalyst for highly selective CO_2 reduction to formate in water/ionic liquid electrolyte

Supplementary Material

Experimental Section

Chemicals

All chemicals including 1-ethyl-3-methylimidazolium tetrafluoroborate (98%), [EMIM](BF_4), tetrabutylammonium tetrafluoroborate, n-Bu_4BF_4, (99%), $CuSO_4.5H_2O$ (99.9%), H_2SO_4 99.8% and CH_3CN (99.9%) were purchased from Sigma-Aldrich.

Methods

For the construction of the 3D Cu nanodendritic porous network, a solution of 0.2 M $CuSO_4$, 1.5 M H_2SO_4 was initially prepared. Then, a Cu plate electrode (1 cm^2) was immersed into the solution and a current of 0.5 A was applied using a galvanostat. Under these conditions, intense H_2 bubbles were generated resulting in Cu deposition in the form of a porous structure.[1,2,3]

Electrochemical measurements were performed in a three-electrode two-compartment cell using a Bio-logic SP300 potentiostat. Ag/AgCl/3M KCl (hereafter abbreviated as Ag/AgCl) was used as the reference electrode and placed in the same compartment as the working electrode. A platinum counter electrode was placed in a separate compartment connected by a glass-frit and filled with the electrolytic solution. The surface of the working electrode was 1 cm^2. All potential values are given versus the potential of the Fc^+/Fc couple added as an internal standard to the solution after measurement. In MeCN (8% H_2O, 0.1M n-Bu_4BF_4): $E_{1/2}$ (Fc^+/Fc) = 0.42 V vs Ag/AgCl. In [EMIM](BF_4)/H_2O (92/8 v/v): $E_{1/2}$ (Fc^+/Fc) = 0.37V vs Ag/AgCl.

H_2 measurements were performed by gas chromatography on a Shimadzu GC-2014 equipped with a Quadrex column, a Thermal Conductivity Detector and using N2 as a carrier gas. Carbon monoxide, methane and other volatile hydrocarbons from the gas phase were analyzed using a gas chromatograph (Shimadzu GC-2010) equipped with a methanizer, a flame induction detector (FID) and a shincarbon ST (Restek) column. Methanol was assayed

by gas chromatography (Shimadzu GC 2010) using an Rtx-1 column (Restek) and a flame induction detector (FID). Formate, oxalate and glyoxylate concentrations were determined by ionic exchange chromatography (883 Basic IC, Metrohm).

^{13}C-formic acid analysis was carried out by ^{13}C-NMR spectroscopy. Electrolysis using a modified Cu electrode was carried out in [EMIM](BF_4)/H_2O (92/8% v/v) under $^{13}CO_2$ saturation. After 2 h, formic acid was analyzed by ^{13}C-NMR spectroscopy after addition of 0.2 ml of CD_3CN to 0.8 ml of the electrolysis solution. A blank experiment with $^{12}CO_2$ was also carried out. For analysis of ^{13}CO a mass spectrometer was directly connected to the electrochemical cell during standard bulk electrolysis under $^{13}CO_2$ saturation. The gas reference was Argon (MW = 40). Gaseous products were then analyzed by mass spectrometry every third minute. Control experiments were also run: (i) electrolysis with an Argon-saturated solution; (ii) electrolysis saturation of non-labelled CO_2.

SEM images were acquired using a Hitachi S-4800 scanning electron microscope. TEM and HRTEM images were obtained on a JEM-2100F transmission electron microscope (JEOL, Japan) with an accelerating voltage of 200 kV.

The X-ray powder diffraction (XRD) patterns were recorded using an X'Pert Pro P analytical diffractometer equipped with either a Cu−Kα radiation source ($\lambda_{K\alpha 1}$ = 1.540598 Å, $\lambda_{K\alpha 2}$ = 1.544426 Å) or a Co−Kα radiation source ($\lambda_{K\alpha 1}$ = 1.78897 Å, $\lambda_{K\alpha 2}$ = 1.79285 Å) with an X'Celerator detector. Rietveld refinements[23] were performed with the Full Prof suite of programs.

Electrochemical diffusion surface area (A_{diff})

The Randles-Sevcik equation served to calculate A_{diff}, the diffusion surface area:[1]

$$i_p = 2.69 \times 10^5 \, n^{3/2} D^{1/2} A_{\text{diff}} C \, \nu^{1/2} \qquad (1)$$

Here, i_p is the peak current corresponding to the reduction of redox species (Fe^{3+}/Fe^{2+}), obtained by CV of a $K_3[Fe(CN)_6]$ solution, n is the number of exchanged electrons, D is the diffusion coefficient of the analyte (7.5×10^{-6} cm^2 s^{-1}),[1] A_{diff} is the diffusional surface area, C ($mol.cm^{-3}$) is the molar concentration of the analyte and ν is the scan rate (V s^{-1}).

CV was recorded using either a Cu plate or a modified Cu electrode in 0.1M phosphate buffer pH 7.0 containing 5mM $K_3[Fe(CN)_6]$ (scan rate $50mV.s^{-1}$). Using the experimental i_p value from the CV, application of the equation above allowed the determination of the A_{diff} value.

Determination of the standard potential of the CO_2/HCOOH couple in CH_3CN

The method used below is directly taken from references [4] and [5] but we reproduce it in full for the sake of clarity. Of note however is the fact that we do not include the inter-liquid junction potential in the value of the standard potential of the CO_2/HCOOH couple versus NHE while we do it in a second stage when we refer it to the reference system used to measure electrochemical potentials.

We first determine the standard potential of the CO_2/HCOOH couple in a solvent S and in the presence of a weak acid AH referred to the aqueous normal hydrogen electrode (NHE). The redox half-reaction reads as follows:

$CO_{2(s)} + 2\ HA_{(s)} + 2\ e^- = HCOOH_{(s)} + 2A^-_{(s)}$

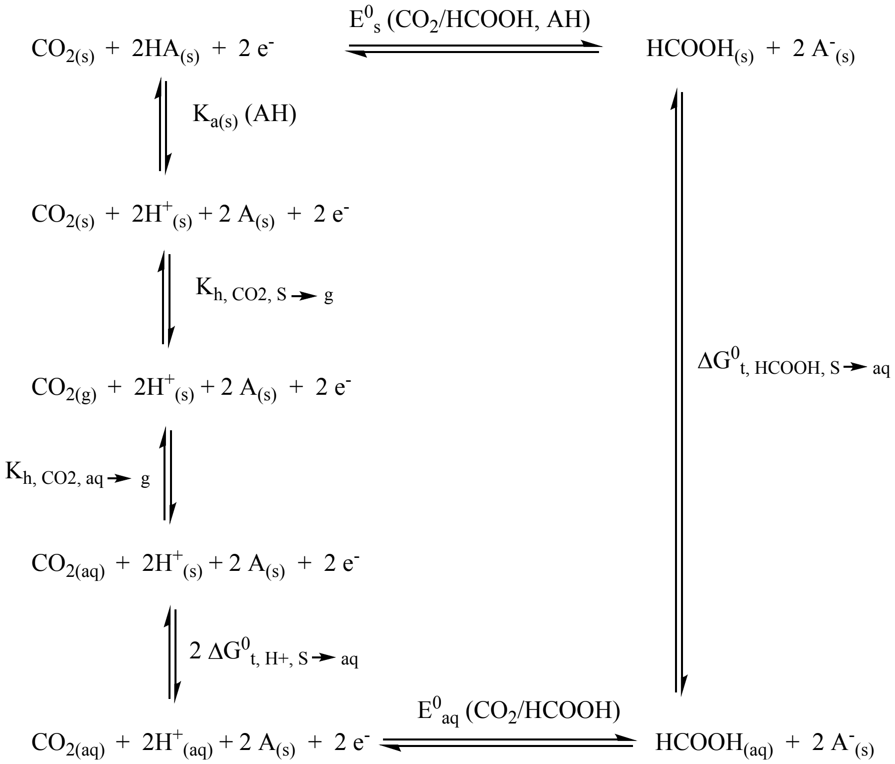

Scheme S1

We use the thermodynamic cycle shown in Scheme S1 and derive the following equation

$$E^0_S(CO_2/HCOOH,AH)=E^0_{aq}(CO_2/HCOOH)-\frac{RTln10}{F}pKa_{(s)}(AH)-\frac{RT}{2F}\ln\left(\frac{K_{h,\ CO_2,aq\to g}}{K_{h,CO_2,S\to g}}\right)-\frac{2\Delta G^0_{t,\ H^+,\ S\to aq}-\Delta G^0_{t,\ HCOOH,\ S\to aq}}{2F}$$

with

$E^0_{aq}(CO_2/HCOOH)$ = –0. 11 V vs NHE at pH 0 [6]

$\Delta G^{0}_{t,\ H^+,\ DMF\to aq} = -46\ kJ/mol$ [7]

$\Delta G^{0}_{t,\ HCOOH,\ DMF\to aq} = -24\ kJ/mol$ [5]

$K_{h,\ CO_2, S\to g} = \frac{P_{CO_2}/P^0}{[CO_2]_{(S)}/C^0}$, with $[CO_2]_{(S)}$ the solubility if CO_2 in the solvent of interest under $P_{CO2} = 10^5$ Pa; $P^0 = 10^5$ Pa and $C^0 = 1$ mol.L^{-1}

$[CO_2]_{CH3CN}$= 0.28mol.L^{-1} [8] and $[CO_2]_{aq}$= 0.038 mol.L^{-1} [9]

We obtain $E^0_{CH3CN}(CO_2/HCOOH, AH)$ = 0.216 V vs NHE – $\frac{RTln10}{F} pKa_{(S)}(AH)$

Considering now that H_2CO_3 formed by hydration of CO_2 is the strongest acid in the CO_2-saturated CH_3CN and using the pKa value of 17.03 previously determined for this couple in CH_3CN,[4] we finally obtain $E^0_{CH3CN}(CO_2/HCOOH, H_2CO_3)$ = –0. 79 V vs NHE.

To refer this potential versus the Fc^+/Fc couple, we use the experimentally determined value of $E(Fc^+/Fc)$ = 0. 42 V vs Ag/AgCl/KCl 3 mol.L^{-1} ($E_{Ag/AgCl}$ = 0.210 V vs NHE) and correct it with the inter-liquid potential (0.100 V)[1] between the aqueous electrolyte of the Ag/AgCl electrode and the CH_3CN solution containing n-Bu_4BF_4 (0.1 mol.L^{-1}). This yields $E_{CH3CN}(Fc^+/Fc)$ = 0. 53 V vs NHE.

Thus **$E^0_{CH3CN}(CO_2/HCOOH, H_2CO_3)$ = –1.32 V vs Fc^+/Fc**

Supporting Figures

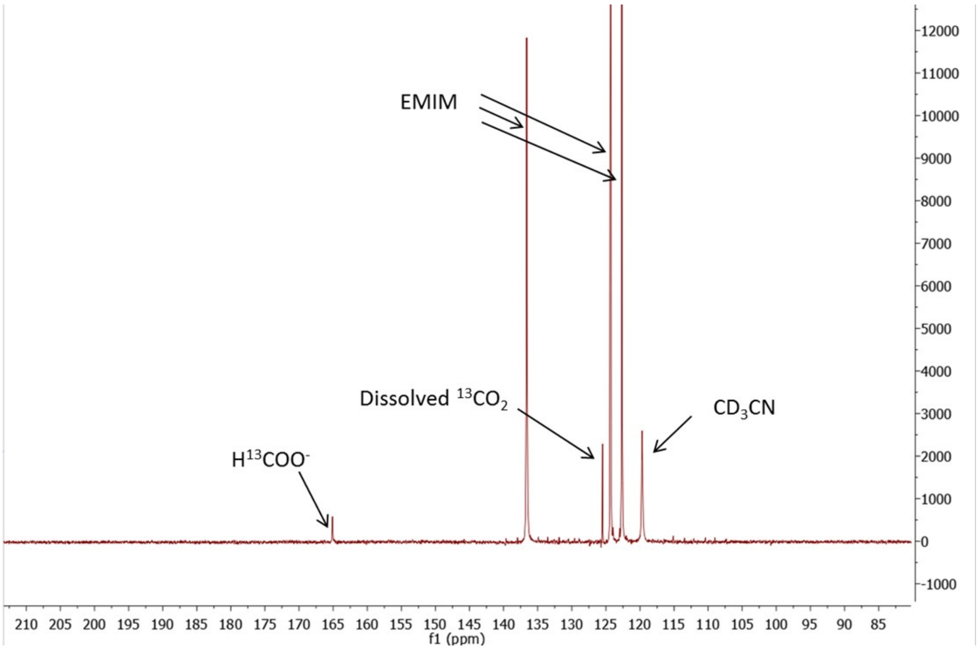

Figure S1: ^{13}C-NMR spectrum of an electrolytic solution using ^{13}CO2 reduction as the substrate in [EMIM](BF_4)/H_2O (92/8 v/v) (0.8ml solution + 0.2ml CD_3CN). ^{13}C-formate is observed at 165 ppm.

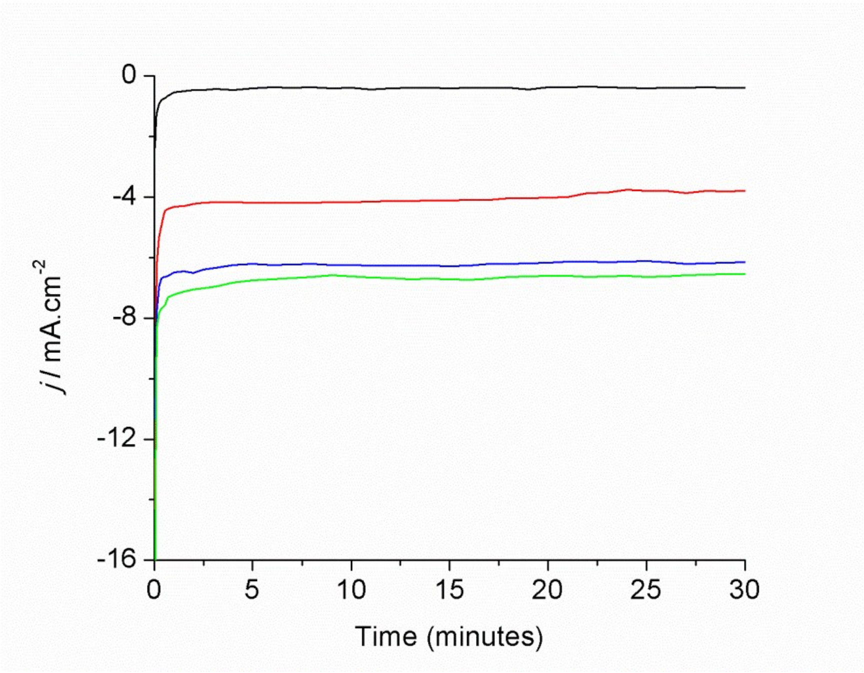

Figure S2: CPE at –1.55V vs Fc^+/Fc in [EMIM](BF_4)/H_2O (92/8 v/v) at CO_2 saturation using the modified Cu electrode obtained after different electrodeposition times: 40s (red), 80s (blue), 120s (green). The modified Cu electrode (80s electrodeposition) was also used under N_2 (black).

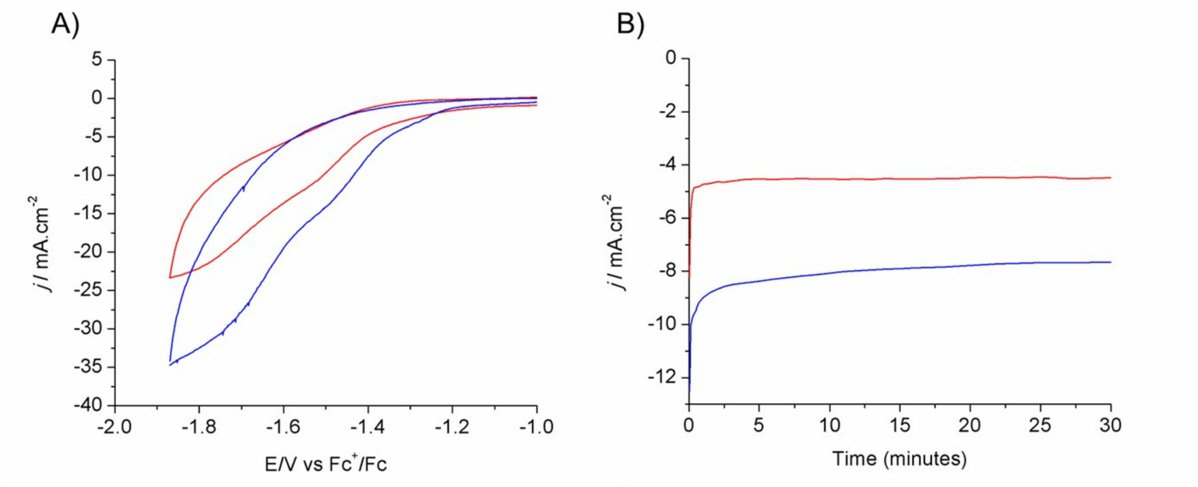

Figure S3: A) Cyclic voltamograms and B) current intensities during CPE using modified Cu electrodes obtained after different electrodeposition times (red: 40s; blue: 80s) in [EMIM](BF_4)/H_2O (85/15 v/v).

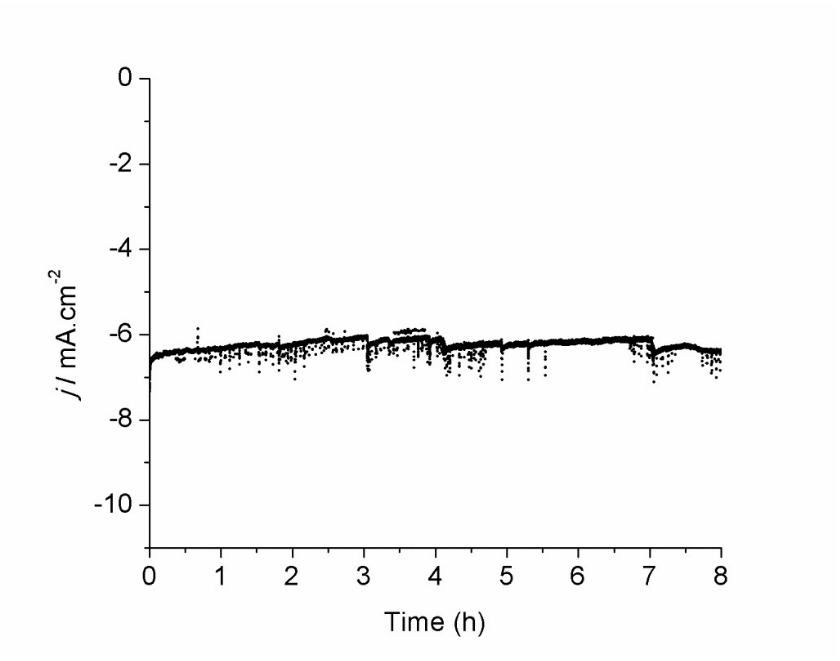

Figure S4: Catalytic current density during 8 h electro-reduction of CO_2 at –1.55 V vs Fc^+/Fc in [EMIM](BF_4)/H_2O (92/8% v/v) solution using a modified Cu electrode (80s electrodeposition).

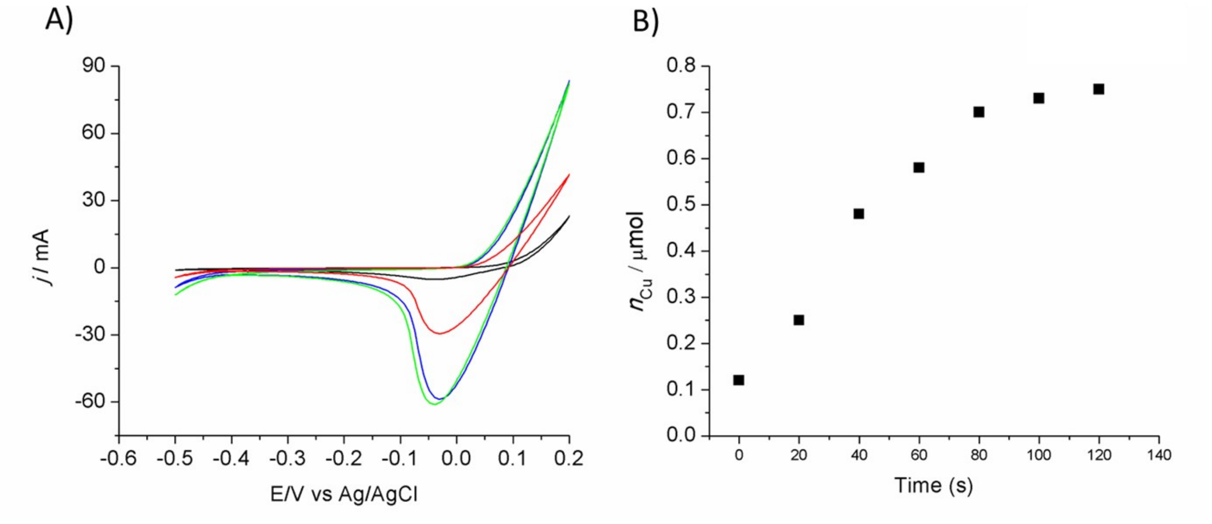

Figure S5: A) Cyclic voltammograms in H_2SO_4 0.5M using a Cu plate (black) or a modified Cu electrode (1 cm^2) obtained after 40s (red), 80s (blue) and 120s (green) electrodeposition. For sake of clarity the data for electrodes obtained after 20, 60 and 100 s are not shown. B) Surface concentration of active Cu calculated from the reduction peak in (A). From each CV total charge Q is calculated, and the amount of active Cu = $Q/(2x1.6x10^{-19}x6.02\ x10^{23})$.

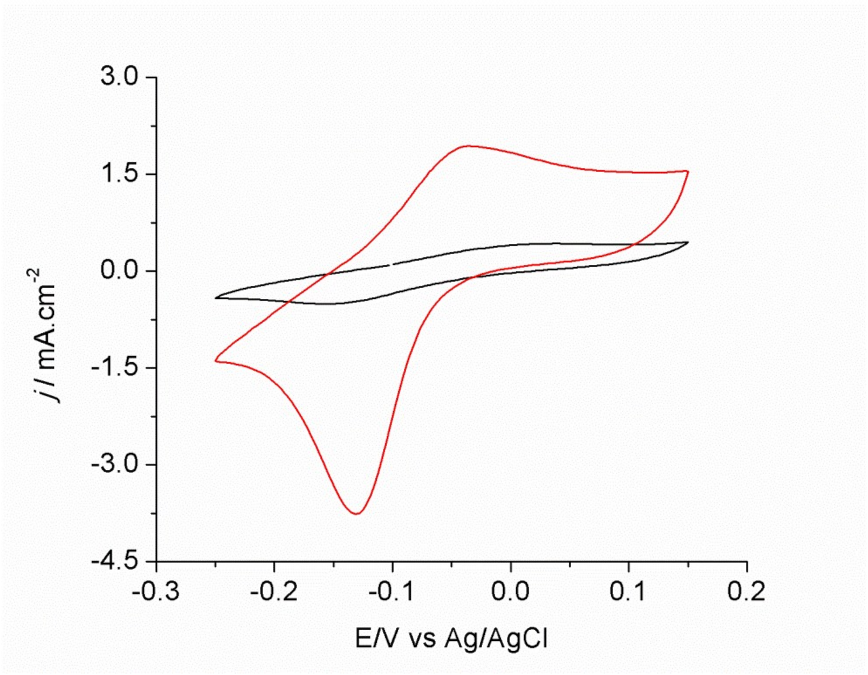

Figure S6: CVs of the Cu plate and the modified Cu electrode (80s deposition) in 0.1M phosphate buffer pH 7.0 containing 5mM $K_3[Fe(CN)_6]$ (scan rate $50mV.s^{-1}$).

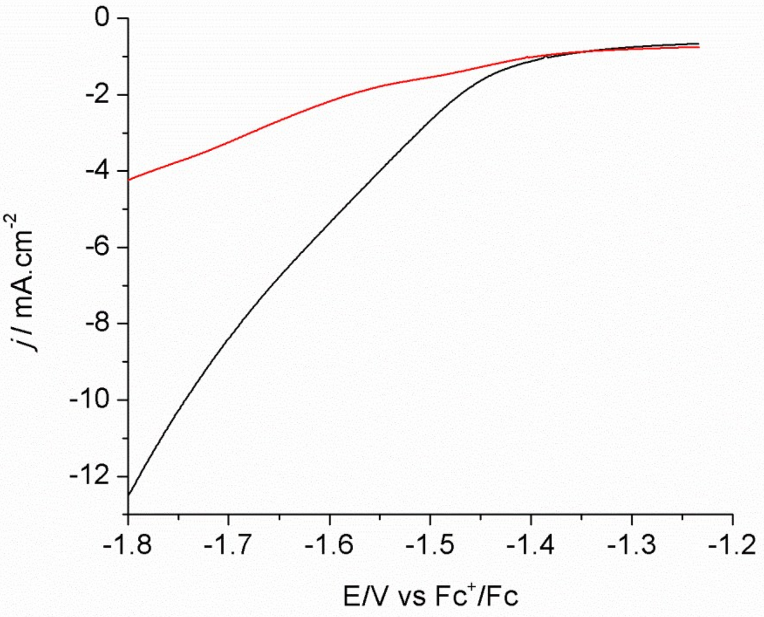

Figure S7: A) LSV of the modified Cu electrode (80s deposition) in MeCN/H_2O (92/8 v/v) + 0.1M n-Bu_4BF_4 under N_2- (red) and CO_2- (black) saturation conditions.

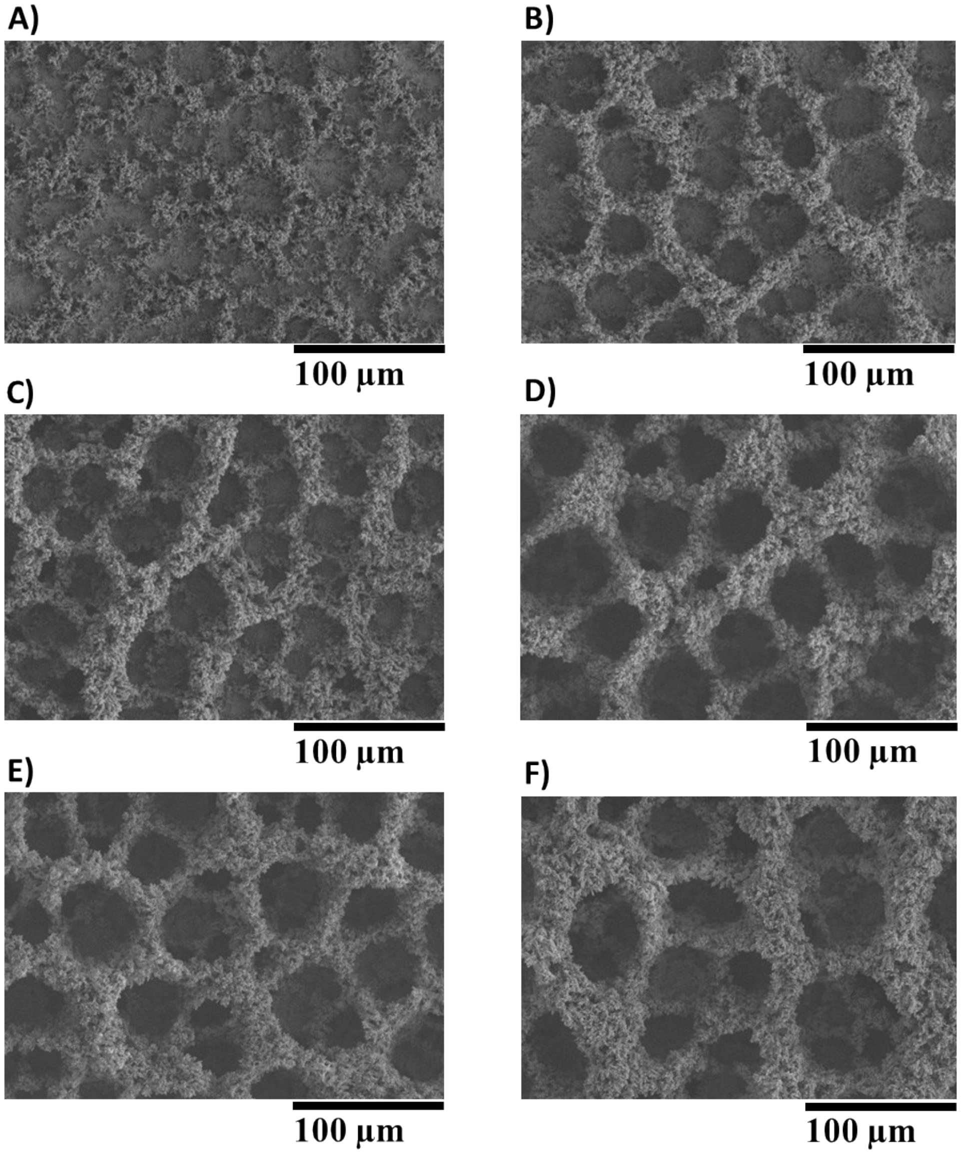

Figure S8: SEM images of the modified Cu electrodes obtained after 20s (A), 40s (B), 60s (C), 80s (D), 120s (E) and 160s (F) electrodeposition.

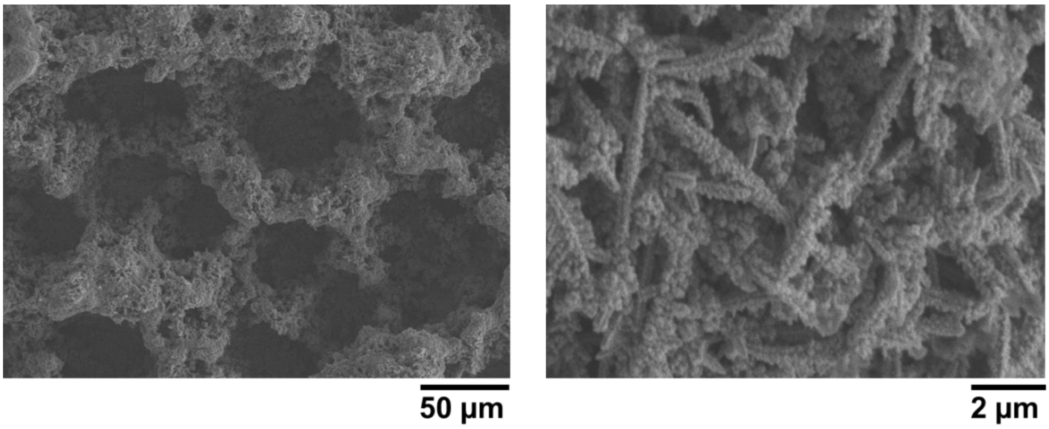

Figure S9: SEM image of the modified Cu electrode (80s electrodeposition) after long-term (8 h) electrolysis.

Table S1. Products and faradic yields during CPE at –1.55V vs Fc^+/Fc under CO_2 saturation in [EMIM](BF_4)/H_2O (92/8 v/v) using a modified Cu electrode obtained after different electrodeposition times

Electrodeposition time	Charge (C)	H_2 (%)	Formate (%)	CO (%)
40s	5.8	9	82	5
80s	8.6	8	83	5
120s	9.1	11	79	6

Table S2: Products and faradic yields during CPE at –1.55 V vs Fc^+/Fc under CO_2 saturation in [EMIM](BF_4)/H_2O (85/15 v/v) using modified Cu electrode obtained after different electrodeposition times.

Electrodeposition time	Charge (C)	H_2 (%)	Formate (%)	CO (%)
40s	10.2	35	50	8
80s	16.8	36	49	9

References:

1. T.N. Huan, T.Ganesha, K.S. Kim, S. Kim, S.H. Han, H. Chung. *Biosens. Bioelectron*. **2011**, *27*, 183-186
2. H.C. Shin, M. Liu, *Chem. Mater*. **2004**, *16*, 5460-5464.
3. C. Zhu, D. Du, A. Eychmuller, Y. Lin, *Chem. Rev*. **2015**, *115*, 8896−8943.
4. C. Costentin, S. Drouet, M. Robert, J.M. Saveant, *Science* **2012**, *338*, 90-94.
5. T.N. Huan, E.S. Andreiadis, J. Heidkamp, P. Simon, E. Derat, S. Cobo, G. Royal, A. Bergmann, P. Strässer, H. Dau, V. Artero, M. Fontecave, *J. Mater. Chem. A* **2015**, *3*, 3901-3907.
6. T. Reda, C.M. Plugge, N.J. Abram, J. Hirst, *Proc. Natl. Acad. Sci. USA* **2008**, *105*, 10654-10658.
7. Y. Marcus, Ion Properties (Marcel Dekker, NY. 1997), p 216.
8. A. Gennaro, A.A. Isse, E. Vianello, *J. Electroanal. Chem.***1990**, *289*, 203-215.
9. D.R. Lide, P.R. Frederikse, Eds. *Handbook of Chemistry and Physics*, *(CRC Press, Boca Raton, FL, ed 76, 1995)*.
